# Roadmap for the accelerated development and clinical translation of fluorescent tracers: Adalimumab-680LT as a proof of concept

**DOI:** 10.1016/j.ijpx.2026.100514

**Published:** 2026-03-10

**Authors:** Henrik K. Huizinga, Rosalie J. van Dijken, Bahez Gareb, Bart G.J. Dekkers, Jacoba van Zanten, Hans H. Westra, Pauline Koopmans, Wouter B. Nagengast, Marjolijn N. Lub-de Hooge

**Affiliations:** a Department of Gastroenterology and Hepatology, University of Groningen, University Medical Center Groningen, Groningen, the Netherlands; b Department of Clinical Pharmacy and Pharmacology, University of Groningen, University Medical Center Groningen, Groningen, the Netherlands

**Keywords:** Fluorescence molecular imaging, Fluorescent tracers, Adalimumab, IRDye 680LT, Roadmap, Clinical translation, GMP production

## Abstract

Fluorescence molecular imaging (FMI), using fluorescent labelled antibodies, can provide insight into local drug distribution, identification of target cells and target engagement. However, clinical translation of novel fluorescent tracers is hampered by their extensive and time-consuming development process, highlighting the need for an accelerated and standardised process. This study presents a general roadmap for accelerated fluorescent tracer development and clinical translation and compares it to the original development process. The accelerated development process introduced feasibility testing, to minimize small-scale experiments, while the introduction of lab runs, combined with a technology transfer, allowed for earlier collection of stability data, further accelerating the development process. Accordingly, adalimumab-680LT was developed as a proof of concept using this accelerated roadmap. Development was mainly performed using labelling parameters, analytical methods, and QC-tests of previously developed tracers and building upon this experience. Adalimumab was successfully conjugated to IRDye 680LT under current Good Manufacturing Practice (cGMP) conditions. Feasibility testing and small-scale experiments yielded a robust and cGMP-compliant production process, with label efficiencies of 82.8 ± 2.9%. Short- and long-term stability was demonstrated in stability studies up to 24 months, with a target binding affinity between 58 and 86%, and a monomer concentration between 0.97 and 1.05 mg/mL. Following the accelerated roadmap, adalimumab-680LT could already be used in phase I/II clinical trials in humans 12 months after the first experiments, a time reduction of 40%, or 8 months. From this point forward, the described roadmap can be applied to develop novel clinical-grade antibody-based fluorescent tracers, saving valuable time and resources.

## Highlights

• Fluorescent tracer development must be accelerated and standardised.
• The novel roadmap enables translation from idea to clinical trial within 12 months.
• Adalimumab-680LT was developed for fluorescent imaging in inflammatory bowel disease.
• Stability of adalimumab-680LT was confirmed up to 24 months after production.
• The first phase I/II clinical trials with adalimumab-680LT are currently ongoing.

## Graphical Abstract

**Figure:**
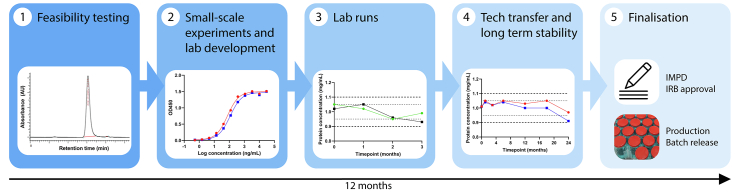

## 1 Introduction

Clinical development of innovative drugs is a time-consuming and expensive process, which can take from less than five years to over twenty years (Brown et al., 2022). In recent years, numerous targeted drug therapies have been developed, including monoclonal antibodies, which are becoming increasingly important in the treatment of various diseases (Kinch et al., 2023). Despite these advancements in the development of and treatment with monoclonal antibodies, challenges persist, including high non-response rates and limited understanding of their distribution and mechanism of action at the target site (Wang et al., 2022; Yanai and Hanauer, 2011).

Fluorescence molecular imaging (FMI), an emerging field in optical imaging, can be used to increase biological and pharmacological insight, and help overcome these challenges (Gabriëls et al., 2024; Tjalma et al., 2020; Nagengast et al., 2019). By administering fluorescent labelled monoclonal antibodies to patients, the drug distribution at the target site of these antibodies can be visualized in real time with specialized cameras during endoscopy or surgery. During procedures, biopsies can be taken and used to determine localisation of the specific antibody target.

In clinical settings, FMI with IRDye labelled tracers has mainly been used with authorised antibodies (Gabriëls et al., 2024; Tjalma et al., 2016; Krishnan et al., 2021; Rosenthal et al., 2015). These tracers are being used for multiple applications. Two recent examples are cetuximab-800CW and vedolizumab-800CW. Cetuximab-800CW was used for intraoperative margin assessment in oral cancer patients, where its added value was demonstrated in identifying tumour-positive surgical margins of oral squamous cell carcinoma (de Wit et al., 2023). Regarding vedolizumab-800CW, FMI was used to study its local drug distribution in patients with inflammatory bowel disease (Gabriëls et al., 2024). This study proved the feasibility of visualizing the distribution and identifying new target cells of vedolizumab using FMI. This highlights the potential of FMI for application during drug development and preclinical trials of novel antibodies, to study the distribution at the target site and unravel the mechanism of action of these novel antibodies.

The process of getting from an antibody to a fluorescent tracer for FMI, and subsequently to the results of a first-in-human clinical trial is extensive, consisting of two main phases. First, the development of a fluorescent antibody complying with the current Good Manufacturing Practice (cGMP) guidelines including preclinical safety testing, when necessary, can take several years to complete. Once finished, the first-in-human safety and efficacy clinical trial can start. These clinical studies can also take up to multiple years to complete, depending on the rate of inclusion. Together this makes the entire process of realising FMI for a single antibody slow and time-consuming, hampering clinical translation. A fast and standardised development process is warranted to accelerate tracer development for FMI using authorised antibodies as well as novel antibodies.

Linssen et al. took the first step in standardisation of the development and clinical translation of optical tracers, described in a roadmap (Linssen et al., 2019). Continued experience with the development of new fluorescent tracers enabled further acceleration of this process. In this paper we demonstrate streamlining of the tracer development process using adalimumab and the fluorescent dye IRDye 680LT. Adalimumab is an antibody targeting tumour necrosis factor (TNF-α), which is used in several inflammatory diseases (Kalliolias and Ivashkiv, 2016). Here we provide a roadmap for accelerated fluorescent tracer development and clinical translation of novel tracers for phase I/II trials, compare it to the original development process and demonstrate the applicability of the accelerated roadmap using adalimumab-680LT as a proof of concept.

## 2 Materials and methods

### 2.1 Development process of adalimumab-680LT

The accelerated development process of adalimumab-680LT consists of five steps. In the first month, feasibility testing was performed to assess labelling of the antibody adalimumab with IRDye 680LT. These experiments were performed using standardised labelling conditions found to be optimal for previously developed tracers, such as a 50 mM sodium phosphate label buffer pH 8.5, a molar dye-to-protein label ratio of 2:1, a conjugation incubation time of 1–2 h and a 50 mM sodium phosphate formulation buffer pH 7.0 (Linssen et al., 2019; Ter Weele et al., 2016; Linssen et al., 2021).

Feasibility testing was followed by further lab development. This included predominantly the development of analytical methods and specifications for Quality Control (QC) testing, and in addition experiments to optimise the labelling process where needed. Although most methods and specifications were adopted from previously developed tracers, the target specific binding assay had to be developed specifically for adalimumab-680LT (Linssen et al., 2019; Ter Weele et al., 2016; Linssen et al., 2021). Final specifications for release and end of shelf-life for adalimumab-680LT were established (Table S1). These specifications are in accordance with current regulations and the European Pharmacopoeia.

Once the optimal labelling conditions were determined and the analytical methods were operational, two lab runs using 15 mg adalimumab were produced and analysed at predetermined time points up to 3 months at 2–8 °C (Table 1). These small-scale batches were produced under comparable conditions as future clinical batches; however, they are produced in the laboratory. Here, most QC-tests were performed to assess the short-term stability of the tracer. After 2–3 months of stability testing, the technology was transferred to the cGMP facility, where a 720 mg full-scale cGMP technology transfer batch was produced for validation of the production process. This production process is identical to the process used for the production of clinical batches. The produced vials of this technology transfer batch are used for a long-term stability study (Table 1).

**Table 1:** Overview of batch size, minimal number of vials, and stability tests for short- and long-term stability.

Lab Run/Short Term Stability					Technology Transfer/Long-term Stability							
**Size**					**Size**							
30 mg					720 mg							
**Vials**					**Vials**							
Lab run 1 (2–8 °C)	3 vials				Refrigerator (2–8 °C)			36 vials				
Lab run 2 (2–8 °C)	3 vials				Room temperature (15–25 °C)			36 vials (18 months)				
					Release testing (*T* = 0)			26 vials				
**Stability testing (months)**					**Stability testing (months)**							
	**0**	**1**	**2**	**3**		**0**	**1**	**3**	**6**	**12**	**18**	**24**
Protein monomer concentration	X	X	X	X	Protein monomer concentration	X	X	X	X	X	X	X
Protein aggregates	X	X	X	X	Protein aggregates	X	X	X	X	X	X	X
Unconjugated IRDye 680LT	X	X	X	X	Unconjugated IRDye 680LT	X	X	X	X	X	X	X
Protein monomer identity	X	X	X	X	Protein monomer identity	X	X	X	X	X	X	X
Protein monomer integrity	X	X	X	X	Protein monomer integrity	X	X	X	X	X	X	X
Target binding affinity	X	X	X	X	Target binding affinity	X	X	X	X	X	X	X
Appearance (turbidity)	X	X	X	X	Appearance (turbidity)	X	X	X	X	X	X	X
Appearance (colour)	X	X	X	X	Appearance (colour)	X	X	X	X	X	X	X
Container closure and label	X	X	X	X	Container closure and label	X	X	X	X	X	X	X
Extractable volume	X	X	X	X	Extractable volume	X	X	X	X	X	X	X
pH	X	X	X	X	pH	X	X	X	X	X	X	X
Osmolality	X	X	X	X	Osmolality	X	X	X	X	X	X	X
Residual solvents (DMSO)					Residual solvents (DMSO)	X						
Bacterial endotoxins					Bacterial endotoxins	X	X	X	X	X	X	X
Sterility					Sterility	X				X		X
Visible Particles	X	X	X	X	Visible Particles	X	X	X	X	X	X	X
Sub-visible Particles					Sub-visible Particles	X				X		X
UV-VIS absorption peaks	X	X	X	X	UV-VIS absorption peaks	X	X	X	X	X	X	X

One month of stability data from the long-term stability study, together with the stability data from the lab runs, is considered sufficient to complete a first version of the quality part of the investigational medicinal product dossier (Q-IMPD) for submission of a phase I/II clinical trial to the institutional review board (IRB) and continue to the production of a clinical batch. During IRB review and the clinical trial itself, additional stability data will become available, which are used to further extend the shelf-life of the tracer and update the Q-IMPD.

The described steps of the accelerated development were performed accordingly for adalimumab-680LT, to confirm the feasibility of the accelerated development process. The steps were summarized in a roadmap and compared to the original development process as described by Linssen et al. (Linssen et al., 2019).

### 2.2 GMP tracer manufacturing

Tracer production procedures were derived from bevacizumab-800CW, cetuximab-800CW, trastuzumab-800CW, vedolizumab-800CW, durvalumab-680LT and nivolumab-800CW, as described previously (Linssen et al., 2019; Ter Weele et al., 2016; Linssen et al., 2021; Huizinga et al., 2025). cGMP production of adalimumab-680LT consisted of buffer exchange, conjugation, purification, and sterile filtration. Briefly, to remove excipients in the solution and to optimise the pH for labelling, adalimumab registered product (Humira®, AbbVie) was buffer exchanged to a 50 mM sodium phosphate buffer pH 8.5 (Apotheek A15, Gorinchem, the Netherlands) using pre-equilibrated PD-10 columns (Cytiva lifesciences, Chicago, IL, USA). cGMP grade IRDye 680LT NHS-ester (LI-COR Biosciences, Lincoln, NE, USA), dissolved in dimethyl sulfoxide (DMSO) (Sigma Aldrich, Darmstadt, Germany) 5 mg/mL, was added to the adalimumab solution in a molar dye-to-protein ratio of 2:1. Hereafter, the solution was incubated protected from light for 1–2 h at room temperature. After the incubation, the solution was purified by another buffer exchange using pre-equilibrated PD-10 columns and formulation buffer (50 mM sodium phosphate and 101 mM sodium chloride, pH 7.0, Apotheek A15, Gorinchem, the Netherlands). The tracer solution was diluted with formulation buffer to a concentration of 1.0 mg/mL. Next, the solution was filtrated into a Flexboy® bag (Sartorius Stedim, Göttingen, Germany), over a sterile 0.2 μm filter which was connected to the bag (Sartopore® 2 gamma filter capsule). Subsequently, aseptic filling of 5.3–5.7 mL tracer over a sterile Millex-GP 0.22 μm filter (Merck KGaA, Darmstadt, Germany) into 10R vials (APG Pharma, Uithoorn, the Netherlands) was performed, after which the vials were closed using a 20 mm bromobutyl rubber stopper and 20 mm aluminium closure (both Datwyler, Altdorf, Switzerland). Closures were crimped using a compressed-air powered semi-automatic crimping tool. Afterwards, vials are labelled, packaged and stored at 2–8 °C, protected from light.

### 2.3 Quality control testing

Table S1 shows the analytical methods and specifications of adalimumab-680LT for release and end of shelf life. Adalimumab-680LT was analysed for label efficiency (LE), protein concentration, soluble protein aggregates, unbound IRDye, protein monomer identity and protein monomer integrity by Size-Exclusion High-Performance Liquid Chromatography (SE-HPLC) using unmodified adalimumab as a standard. Label efficiency is the percentage of dye conjugated to the antibody relative to the total amount of dye, measured at 679 nm. Protein identity is determined by comparing the retention time and protein integrity by comparing the peak shape of adalimumab-680LT with the adalimumab standard. Data was collected by detection with a Diode-Array Detector (DAD). The system consisted of a 5110 Chromaster pump, 5210 Chromaster injector and 5430 Chromaster DAD (Hitachi, Tokyo, Japan), a Biosep SEC S3000 300 × 7.8 mm column (Phenomenex, Torrance, CA, USA), with isocratic elution and a mobile phase of phosphate buffered saline (PBS, NaCl 140.0 mM, Na_2_HPO_4_ 9.0 mM, NaH_2_PO_4_ 1.3 mM) pH 7.5 at a flow of 1.0 mL/min. For every peak in the chromatogram, the UV absorption spectrum from 200 to 900 nm was measured. Peaks were analysed at 280 nm for protein measurements, at 679 nm for LE, and at 676 nm for free IRDye 680LT measurements. The system was operated, and data was analysed with the Chromaster System Manager (CSM) software package (Hitachi, Tokyo, Japan).

General appearance, solution colour, turbidity and visible particles were assessed visually. Osmolality, pH, bacterial endotoxins, bioburden, residual solvents (DMSO) and sub-visible particles were tested according to their respective Ph. Eur. monographs.

Target affinity was assessed by an in-house developed indirect enzyme-linked immunosorbent assay (ELISA). ELISA set-up and design was performed like described in previous protocols (Linssen et al., 2019; Huizinga et al., 2025), with the only differences being the antigen used, recombinant TNF-α (Sino Biologicals Europe GmbH, Eschborn, Germany) (1.0 μg/mL), and the dilution series (30 μg/mL to 0.5 ng/mL) of both the commercially available adalimumab and adalimumab-680LT. A 4-parameter logistic regression was performed on the yielded data to fit a sigmoidal curve. From the sigmoidal fit, the EC_50_ value was calculated. Target affinity was calculated by comparing the EC_50_ of adalimumab-680LT and the reference adalimumab.

SE-HPLC- and ELISA-methods were validated according to ICH Q2 guidelines (Tables S2–3). All other tests were validated according to their respective Ph. Eur. monographs.

## 3 Results

### 3.1 Accelerated roadmap

**Fig. 1:**
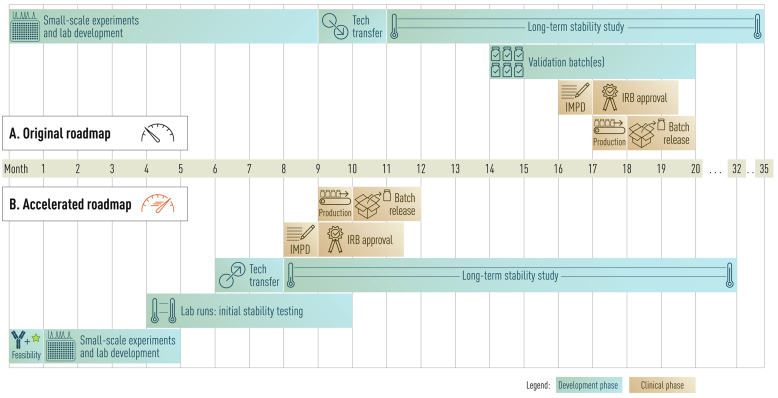
**Comparison of the original development process and accelerated roadmap for fluorescent tracer development.** (A) The original development process consists of four development phases, taking 20 months for the development and release of a clinical batch of a novel fluorescent tracer (12). (B) In the accelerated roadmap five development phases are defined, leading to the production and release of a clinical batch of a novel fluorescent tracer within 12 months.

A comparison between the original development process and the accelerated roadmap is depicted in Fig. 1, where a timeline of the different phases of both development processes is displayed. The original development process divides the development process in four phases: small-scale experiments and lab development, technology transfer, long-term stability study and validation batches. On average, the first phase required nine months to complete. Next, a full-scale cGMP batch was produced during technology transfer, to evaluate the production process. These produced vials were used for a long-term stability study. Finally, two validation batches were produced, followed by the clinical production and batch release of the fluorescent tracer. This whole process took approximately twenty months to complete.

The experience we obtained in fluorescent tracer production over the years enabled adaptation and shortening of the development process to five phases; feasibility testing, small-scale experiments and lab development, lab runs, technology transfer and a long-term stability study. Two substantial adaptations were made to achieve the major time reduction of the accelerated roadmap. First, we implemented one month of feasibility testing in which standardised conditions were tested. Consequently, the conduction of the small-scale experiments and lab development was shortened from nine to four months. The implementation of this step reduced the need for optimalisation of most production and analytical methods, hereby saving time in the entire development process.

Second, the process of collecting stability data was optimised. Originally, the first stability data from the technology transfer batch were collected after eleven months. A few months later, multiple validation batches were produced to validate the production process and get more data on tracer stability. This process took at least six months after the production of the technology transfer batch. However, the introduction of lab runs allowed for earlier collection of stability data, with the first stability data already collected after four months. The technology transfer could also be performed at an earlier stage (month 7–8) and combined with the data from the lab runs one month of long-term stability data is sufficient to draft a Q-IMPD. Furthermore, the technology transfer served as a validation batch for the production process developed in the lab. As a result, the transition from lab development to technology transfer to drafting an IMPD became more efficient.

The described changes resulted in an optimised and standardised five-phase roadmap that can deliver a tracer for clinical use within a year. Compared to the original development process as well as typical tracer development processes, the accelerated roadmap has a fast development phase and an efficient transition from stability studies to requesting IRB approval and the start of a clinical trial. The development of adalimumab-680LT was performed according to the accelerated roadmap. The results of the different phases of this development process for adalimumab-680LT are described below.

### 3.2 Feasibility testing (phase I)

**Fig. 2:**
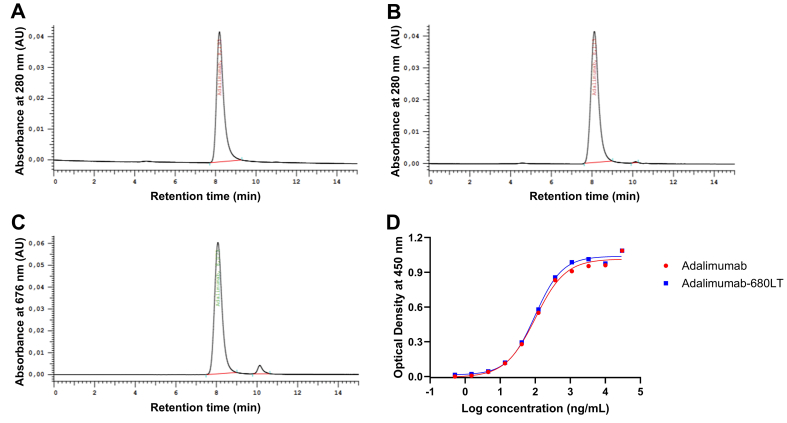
**Results for the feasibility testing and lab development of adalimumab-680LT.** SE-HPLC chromatograms of (A) unmodified adalimumab and (B) adalimumab-680LT at 280 nm, (C) chromatogram of adalimumab-680LT and free IRDye 680LT at 676 nm and (D) results of the indirect ELISA of unmodified adalimumab and adalimumab-680LT.

Feasibility testing demonstrated that it was feasible to label adalimumab with IRDye 680LT. Chromatograms of adalimumab-680LT (Fig. 2B) were comparable to the chromatograms of unmodified adalimumab (Fig. 2A), with a similar peak shape and retention time, confirming monomer integrity and identity. The amount of free dye was determined at 676 nm and was 1.38 ± 0.76%. An example of the free dye at 676 nm is shown in Fig. 2C. Label efficiency proved adequate with efficiencies of 82.8 ± 2.9%.

### 3.3 Small-scale experiments and lab development (phase II)

The next phase on the roadmap was lab development. Besides optimising the labelling of adalimumab-680LT, lab development was also used to develop analytical methods and establish corresponding QC-specifications. An indirect ELISA was developed to determine the binding affinity of adalimumab-680LT to its target TNF-α and compare this to the affinity of unmodified adalimumab. Fig. 2D shows the sigmoidal curves of both adalimumab-680LT and unmodified adalimumab, resulting in a binding affinity of 102%.

### 3.4 Lab runs (phase III)

**Fig. 3:**
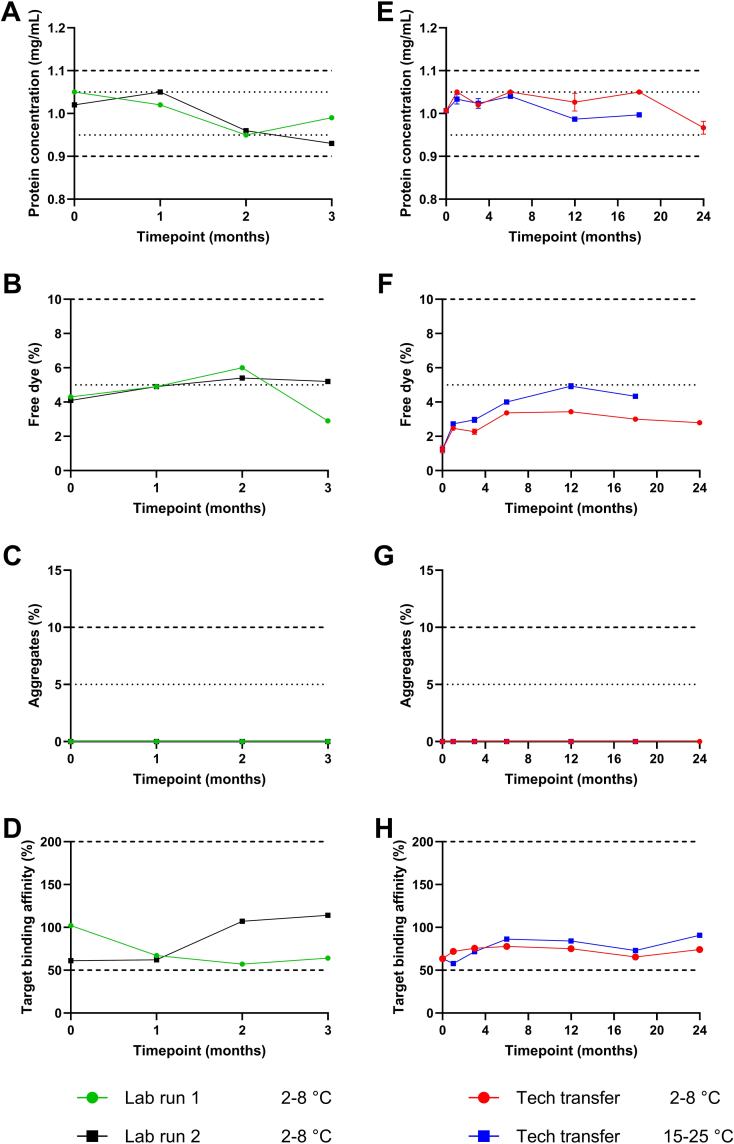
**Stability results of adalimumab-680LT.** Release specifications are displayed with dotted lines, and end of shelf-life specifications are displayed with dashed lines. (A) Protein concentration, (B) percentage of free dye, (C) percentage of aggregates, and (D) target binding affinity of lab run 1 and 2 were tested during 3 months. For the technology transfer batch, (E) protein concentration, (F) percentage of free dye, (G) percentage of aggregates and (H) target binding affinity were tested at two different temperatures during 18 or 24 months. The stability study of the technology transfer batch at 2–8 °C is still ongoing. (A-C) are means of two different measurements, (*E*-G) are means + standard deviations of three different measurements, and (D and H) are means of two measurements.

To assess the short-term stability of adalimumab-680LT, two lab runs were performed. Fig. 3A-D presents the results after 3 months of stability testing. Both the protein concentration and the amount of free dye met the release specifications (Table S1) and remained within the end of shelf-life specification during the entire duration of the lab run. No aggregates were detected at any time point. Although there was some variability in target binding affinity between the two lab runs and at different time points, all measurements were within the specification of 50–200% (Table S1). Together, these results show that adalimumab-680LT is stable for at least 3 months since all parameters remained within specification during the entire duration of the lab run.

### 3.5 Technology transfer and long-term stability study (phase IV and V)

The final part of tracer development was performing the technology transfer under cGMP conditions to validate the production process and initiate the long-term stability study. Table S1 provides all product specifications at release and at end-of-shelf life and their respective methods for testing. The produced batch of adalimumab-680LT vials was used for a long-term stability study at according to the ICH Q1A guideline. Vials were stored at the intended temperature of 2–8 °C and at 15–25 °C to account for potential temperature excursions during storage of clinical batches (Table 1). Fig. 3E-H provides the results obtained after the first 18 (15–25 °C) and 24 (2–8 °C) months of this ongoing stability study. During 24 months storage at 2–8 °C and 18 months at 15–25 °C, monomer concentrations between 0.97 and 1.05 mg/mL were measured. Target binding affinities between 58 and 86% were measured. Similar to the lab runs, all stability results of adalimumab-680LT complied with the release specifications and remained within the end-of shelf-life specifications (Tables S4–5). This demonstrates the stability of adalimumab-680LT during at least 24 months.

## 4 Discussion

This paper presents a roadmap for the accelerated development of fluorescent tracers and demonstrates the practicability of this roadmap using adalimumab-680LT as a proof of concept. The presented roadmap enables faster transition from stability studies to requesting IRB approval and the start of a clinical trial, compared to the original development process (Linssen et al., 2019).

There are two main contributors to the significant time reduction of the accelerated roadmap. The first contributor is the implementation of feasibility testing, to assess within one month if an antibody can indeed be labelled with standardised labelling conditions. Using the labelling protocol described in this paper, any therapeutic antibody can undergo the feasibility testing. SE-HPLC-methods have been set up for previously developed IRDye 680LT- and IRDye 800CW-based tracers and can be used to analyse the potential new tracer on label efficiency, tracer concentration, free IRDye and aggregate formation (Linssen et al., 2019; Ter Weele et al., 2016; Linssen et al., 2021; Huizinga et al., 2025). During SE-HPLC-analysis of the novel tracer, it will become clear if the tracer was labelled successfully. This is mainly based on retention time and peak integrity. If the chromatography of the new tracer displays split main peaks, shoulders, heavy fronting or tailing, or if the main peaks of the chromatograms at 280 nm and 676/775 nm do not overlap, it is likely that the antibody cannot be labelled. Only if the labelling was successful, or if the labelling needs minor optimalisation, the next development steps can be taken. This creates a clear go/no go cutoff point during feasibility testing. In the case of adalimumab-680LT, feasibility testing showed promising results. Therefore, the development process could be continued to small scale experiments and lab development.

Originally, the stage of small-scale experiments and lab development was used to perform multiple experiments to characterise and optimise the conjugation of the antibody with IRDye, which was deemed necessary at that time to characterise the tracers (Linssen et al., 2019; Ter Weele et al., 2016; Linssen et al., 2021). However, extensive experience with tracer development processes and QC-testing enabled a selective and risk-based approach to perform experiments in the development phase of the accelerated roadmap to only get the essential results needed for clinical translation. This includes the set-up of an indirect ELISA-assay, to assess the target binding affinity of a novel tracer. Although this QC-test has to be set up for every new tracer, the ELISA-protocol as described in this paper can be used as a template for this, changing only the relevant reagents and concentrations.

Together, this shortens the lab development from nine to four months and as exemplified with adalimimab-680LT this highly accelerated development phase is both feasible and acceptable.

The second contributor to the major time reduction of the accelerated roadmap is the earlier generation of stability data with the introduction of lab runs. By introducing lab runs in the accelerated process, at least four months can be cut from the original development process, whilst still collecting sufficient stability data. Although the shelf-life of the tracer is based on the stability data from the long-term stability batch, the lab runs can give a good indication on the expected stability.

Since most authorised antibodies have a long shelf-life, the tracer shelf-life is expected to be up to multiple years. For example, Humira® (adalimumab, AbbVie) has a shelf-life of two years (AbbVie, 2024). Moreover, published and unpublished data on IRDye-based tracers provide a wealth of information and increasing knowledge on the expected stability of tracer products.

In 2014, Bhattacharyya et al. demonstrated that panitumumab-800CW remained stable for ten days (Bhattacharyya et al., 2014). In 2016, Ter Weele et al. showed the stability of bevacizumab-800CW for three months, which Linssen et al. also proved for both cetuximab-800CW and trastuzumab-800CW (Linssen et al., 2019; Ter Weele et al., 2016). Most recent, Huizinga et al. published twelve-month stability data on durvalumab-680LT and nivolumab-800CW (Huizinga et al., 2025). Finally, this paper demonstrates a more extended stability of fluorescent tracers, with 24 months stability of adalimumab-680LT at 2–8 °C. Unpublished data on different IRDye based tracers have even shown stability of up to four years. With this combined knowledge on both antibody and tracer stability, it is acceptable to accelerate the transition from technology transfer to the production and release of a clinical batch without awaiting the long-term results of the stability study.

Besides being a long-term stability batch, the technology transfer batch also serves as a validation batch for the production process developed in the lab. Originally, multiple validation batches were produced to validate the production process (Linssen et al., 2019). However, with the introduction of lab runs, the production process is already evaluated in a small scale on the lab. Furthermore, the production process is similar for most fluorescent tracer and extensive experience was already obtained on the cGMP production of fluorescent tracers (Linssen et al., 2019; Ter Weele et al., 2016; Huizinga et al., 2025). Therefore, one technology transfer batch of a new tracer product is sufficient to validate the cGMP production process for phase I/II clinical trials, saving time and money.

Initially, a toxicity study had to be conducted for new tracer products. The roadmap of Linssen et al. also described a toxicity study of both the used dye and the new antibody-dye conjugate (Linssen et al., 2019). However, toxicity studies have been performed, and safety has been demonstrated for both IRDye 800CW and IRDye 680LT (Huizinga et al., 2025; Marshall et al., 2010). Moreover, safety of authorised antibodies is extensively described in their respective European Public Assessment Reports published by the European Medicine Agency. Toxicity studies would only be necessary if new dyes are used for tracer development, or if compounds are used of which no phase I/II clinical safety data is available. Since previous toxicity studies of adalimumab, IRDye 680LT and previous IRDye-based tracer products substantiate the safety of the developed product, we omitted this step from the accelerated roadmap and thus from the development process of adalimumab-680LT (Huizinga et al., 2025; AbbVie, 2003).

Although this accelerated roadmap facilitates faster fluorescent tracer development, some limitations remain. Importantly, some key data, such as initial safety data and target binding affinity of the antibody, must be available before the fluorescent tracer development process can be accelerated. For authorised antibodies these data are available. However, if these data are not yet available for non-authorised antibodies, they should be gathered before proceeding with fluorescent tracer development. It is also of note that optimal application of the accelerated roadmap can only be performed if the necessary facilities and resources are present. However, this limitation is also applicable to fluorescent tracer development as a whole, including development following the original roadmap (Linssen et al., 2019).

Together, the accelerated roadmap makes it possible to start clinical production and submission to the IRB for approval in month 10 compared to month 18 in the original development process. This results in a clinical batch of tracer, ready for use within 12 months, compared to the original 20 months, a time reduction of 40%. This is exemplified with the development and clinical translation of adalimumab-680LT, which could be used after 12 months in a phase I/II clinical trial in humans (NCT06117423).

## 5 Conclusions

The presented roadmap is a major step towards a fast and standardised development process of antibody based fluorescent tracers. The roadmap can be used as a guideline in fluorescent tracer development for all authorised antibodies and novel antibodies with phase I safety data available. However, the guideline is not tailor-made. For example, if feasibility testing with the initial labelling parameters does not give satisfactory results, a longer and more extensive development process could still be necessary. Furthermore, the roadmap can also be applied during drug development programs; however, assembling the required documentation might take some more time. Although these exceptions would extend the timeline, the roadmap can still be used as a guideline to streamline and speed up the development process. Therefore, with this roadmap, the transition from bench-to-bedside with fluorescent labelled antibodies has been significantly accelerated, streamlining the development process and thereby saving valuable time and resources.

## CRediT authorship contribution statement

**Henrik K. Huizinga:** Writing – review & editing, Writing – original draft, Visualization, Validation, Project administration, Methodology, Investigation, Formal analysis. **Rosalie J. van Dijken:** Writing – review & editing, Writing – original draft, Visualization, Validation, Project administration, Methodology, Investigation, Formal analysis. **Bahez Gareb:** Writing – review & editing, Resources, Methodology. **Bart G.J. Dekkers:** Writing – review & editing, Resources, Methodology, Conceptualization. **Jacoba van Zanten:** Writing – review & editing, Resources, Methodology. **Hans H. Westra:** Writing – review & editing, Validation, Investigation. **Pauline Koopmans:** Writing – review & editing, Validation, Investigation. **Wouter B. Nagengast:** Writing – review & editing, Supervision, Methodology, Funding acquisition, Conceptualization. **Marjolijn N. Lub-de Hooge:** Writing – review & editing, Supervision, Resources, Methodology, Funding acquisition, Conceptualization.

## Declaration of competing interest

The authors declare the following financial interests/personal relationships which may be considered as potential competing interests:

Marjolijn Lub-de Hooge reports financial support was provided by Horizon Europe. Wouter Nagengast reports financial support was provided by Horizon Europe. If there are other authors, they declare that they have no known competing financial interests or personal relationships that could have appeared to influence the work reported in this paper.

## Data Availability

Data will be made available on request.

## Appendix A Supplementary data

Supplementary material includes product specifications, validation data and detailed stability data. Image 1

## References

[bb0005] AbbVie (2003).

[bb0010] AbbVie (2024).

[bb0015] Bhattacharyya S. (2014). Synthesis and biological evaluation of panitumumab-IRDye800 conjugate as a fluorescence imaging probe for EGFR-expressing cancers. Medchemcomm.

[bb0020] Brown D.G., Wobst H.J., Kapoor A., Kenna L.A., Southall N. (2022). Clinical development times for innovative drugs. Nat. Rev. Drug Discov..

[bb0025] de Wit J.G. (2023). EGFR-targeted fluorescence molecular imaging for intraoperative margin assessment in oral cancer patients: a phase II trial. Nat. Commun..

[bb0030] Gabriëls R.Y. (2024). Fluorescently labelled vedolizumab to visualise drug distribution and mucosal target cells in inflammatory bowel disease. Gut.

[bb0035] Huizinga H.K. (2025). Development of clinical-grade Durvalumab-680LT and Nivolumab-800CW for multispectral fluorescent imaging of the PD-1/PD-L1 axis of the immune checkpoint pathway. Pharmaceuticals.

[bb0040] Kalliolias G.D., Ivashkiv L.B. (2016). TNF biology, pathogenic mechanisms and emerging therapeutic strategies. Nat. Rev. Rheumatol..

[bb0045] Kinch M.S., Kraft Z., Schwartz T. (2023). Monoclonal antibodies: Trends in therapeutic success and commercial focus. Drug Discov. Today.

[bb0050] Krishnan G. (2021). Metastatic and sentinel lymph node mapping using intravenously delivered Panitumumab-IRDye800CW. Theranostics.

[bb0055] Linssen M.D. (2019). Roadmap for the development and clinical translation of optical tracers cetuximab-800CW and trastuzumab-800CW. J. Nucl. Med..

[bb0060] Linssen M.D. (2021). Development and characterisation of antibody-based optical imaging probes for inflammatory bowel disease. Pharmaceuticals.

[bb0065] Marshall M.V., Draney D., Sevick-Muraca E.M., Olive D.M. (2010). Single-dose intravenous toxicity study of IRDye 800CW in Sprague-dawley Rats. Mol. Imaging Biol..

[bb0070] Nagengast W.B. (2019). Near-infrared fluorescence molecular endoscopy detects dysplastic oesophageal lesions using topical and systemic tracer of vascular endothelial growth factor A. Gut.

[bb0075] Rosenthal E.L. (2015). Safety and tumor specificity of cetuximab-IRDye800 for surgical navigation in head and neck cancer. Clin. Cancer Res..

[bb0080] Ter Weele E.J. (2016). Development, preclinical safety, formulation, and stability of clinical grade bevacizumab-800CW, a new near infrared fluorescent imaging agent for first in human use. Eur. J. Pharm. Biopharm..

[bb0085] Tjalma J.J. (2016). Molecular fluorescence endoscopy targeting vascular endothelial growth factor a for improved colorectal polyp detection. J. Nucl. Med..

[bb0090] Tjalma J.J.J. (2020). Quantitative fluorescence endoscopy: an innovative endoscopy approach to evaluate neoadjuvant treatment response in locally advanced rectal cancer. Gut.

[bb0095] Wang D.R., Wu X.L., Sun Y.L. (2022). Therapeutic targets and biomarkers of tumor immunotherapy: response versus non-response. Signal Transduct. Target. Ther..

[bb0100] Yanai H., Hanauer S.B. (2011). Assessing response and loss of response to biological therapies in IBD. Am. J. Gastroenterol..

