# Supplementary material for: Roadmap for the accelerated development and clinical translation of fluorescent tracers: Adalimumab-680LT as a proof of concept
Source: Int J Pharm X. 2026 Mar 10;11:100514. doi: 10.1016/j.ijpx.2026.100514 (PMC13000525; doi:10.1016/j.ijpx.2026.100514)
Supplement: Supplementary file 1 — Supplementary material includes product specifications, validation data and detailed stability data. [file mmc1.docx]

**Supplementary data**

**Table S1: Product specifications for adalimumab-680LT (1.0 mg/mL, 5.0 mL) at release and end of shelf life.**

| Test | Method | Specification release | Specification end of shelf life |
| --- | --- | --- | --- |
| Protein monomer concentration | SE-HPLC | 0.95 – 1.05 mg/mL | 0.90 – 1.10 mg/mL |
| Protein aggregates | SE-HPLC | ≤5.0% | ≤10% |
| Unconjugated IRDye 680LT | SE-HPLC | ≤5.0% | ≤10% |
| Protein monomer identity | SE-HPLC | Retention time comparable to reference standard | Retention time comparable to reference standard |
| Protein monomer integrity | SE-HPLC | Peak shape comparable to reference standard; no shoulders, minimal tailing | Peak shape comparable to reference standard; no shoulders, minimal tailing |
| UV-VIS absorption peaks | SE-HPLC | Peaks at 280±3 nm and 676±3 nm. | Peaks at 280±3 nm and 676±3 nm. |
| Target binding affinity | Indirect ELISA | 50-200% | 50-200% |
| Appearance (turbidity) | Visual inspection | Clear to slightly opalescent solution | Clear to slightly opalescent solution |
| Appearance (colour) | Visual comparison to colour standards | Colour tone: comparable to reference | Colour tone: comparable to reference |
| Container closure and label | Visual inspection | Closure intact, label legible and intact | Closure intact, label legible and intact |
| Extractable volume | Ph. Eur. 2.9.17 | ≥5.0 mL | ≥5.0 mL |
| pH | Ph. Eur. 2.2.3 | 6.9 – 7.1 | 6.9 – 7.1 |
| Osmolality | Ph. Eur. 2.2.35 | 270 - 310 mOsmol/kg | 270 - 310 mOsmol/kg |
| Residual solvents (DMSO) | Ph. Eur. 2.4.24  Ph. Eur. 5.4 | ≤50.0 mg/L | NA |
| Bacterial endotoxins | Ph. Eur. 2.6.14 | ≤5.0 EU/mL | ≤5.0 EU/mL |
| Sterility | Ph. Eur. 2.6.1 | Sterile | Sterile |
| Visible Particles | Ph. Eur. 2.9.20 | Practically free of visible particles | Practically free of visible particles |
| Sub-visible Particles | Ph. Eur. 2.9.19 | Particles ≥ 10 μm ≤ 6000/vial  Particles ≥ 25 μm ≤ 600/vial | Particles ≥ 10 μm ≤ 6000/vial  Particles ≥ 25 μm ≤ 600/vial |

Table S2: SE-HPLC validation results of adalimumab

| **Precision and accuracy** | | | | |
| --- | --- | --- | --- | --- |
|  | **Specification** | **LOW**  **250 mg/L** | **MED**  **625 mg/L** | **HIGH**  **1250 mg/L** |
| Within CV (%) | ≤ 10% | 2.0 | 0.5 | 0.4 |
| Between CV (%) | ≤ 10% | 3.1 | 0.0 | 1.4 |
| Total CV (%) | ≤ 10% | 3.7 | 0.5 | 1.5 |
| Bias (%) | ≤ 10% | -3.0 | 1.4 | -0.5 |
|  | | | | |
| **Linearity (correlation coefficient)** | | 0.9988 | | |
| **Range** | | 100-1500 mg/L | | |
| **Specificity/Selectivity** | | No interfering peaks | | |
| **Limit of quantification** | | 100 mg/L | | |

Table S3: ELISA validation results of adalimumab

| **Precision** | | | | |
| --- | --- | --- | --- | --- |
|  | **Specification** | **LOW**  **4.6 ng/mL** | **MED**  **370 ng/mL** | **HIGH**  **30 000 ng/mL** |
| Repeatability (%) | ≤ 15% | 5.3 | 1.3 | 0.9 |
| Intermediate precision (%) | ≤ 15% | 14.8 | 9.9 | 12.6 |
|  | | | | |
| **Range** | | 0.5-30 000 ng/mL | | |
| **Linearity (correlation coefficient)** | | 0.995 | | |
| **Specificity/Selectivity** | | Signal of non-target antibodies is ≤10% of the average signal of adalimumab | | |
| **Limit of detection** | | 0.5 ng/mL | | |
| **Limit of quantification** | | 4.6 ng/mL | | |

**Table S4: Stability results of adalimumab-680LT technology transfer and stability batch after 24 months storage at 2-8** °**C. All tests marked with a star (*) were performed in triplicate; the mean value for each time point is shown. Of the triplicate measurements, none of the individual results fell outside the predefined specifications. All other tests were performed as single measurement or according to the applicable European Pharmacopeia method.**

|  | |  | **Test moments (months)** | | | |  | | | |
| --- | --- | --- | --- | --- | --- | --- | --- | --- | --- | --- |
| **Test** | **Specification** | | **0** | **1** | **3** | **6** | | **12** | **18** | **24** |
| Protein monomer concentration * | Release: 0.95 – 1.05 mg/mL  Shelf-life: 0.90 – 1.10 mg/mL | | 1.01 mg/mL | 1.05 mg/mL | 1.02 mg/mL | 1.05 mg/mL | | 1.03 mg/mL | 1.05 mg/mL | 0.97 mg/mL |
| Protein aggregates * | Release: ≤5.0%  Shelf-life: ≤10% | | N.D. | N.D. | N.D. | N.D. | | N.D. | N.D. | N.D. |
| Unconjugated IRDye 680LT * | Release: ≤5.0%  Shelf-life: ≤10% | | 1.2% | 2.5% | 2.3% | 3.3% | | 3.4% | 3.0% | 2.8% |
| Protein monomer identity * | Retention time comparable to reference standard | | Conform | Conform | Conform | Conform | | Conform | Conform | Conform |
| Protein monomer integrity * | Peak shape comparable to reference standard; no shoulders, minimal tailing | | Conform | Conform | Conform | Conform | | Conform | Conform | Conform |
| Target binding affinity | 50-200% | | 63% | 72% | 76% | 78% | | 75% | 65% | 74% |
| Appearance (turbidity) * | Clear to slightly opalescent solution | | Conform | Conform | Conform | Conform | | Conform | Conform | Conform |
| Appearance (colour) * | For information | | Ref 5 | Ref 5 | Ref 5 | Ref 5 | | Ref 5 | Ref 5 | Ref 5 |
| Container closure and label * | Closure intact, label legible and intact. | | Conform | Conform | Conform | Conform | | Conform | Conform | Conform |
| Extractable volume * | ≥5.0 mL | | 5.1 mL | 5.1 mL | 5.1 mL | 5.1 mL | | 5.0 mL | 5.1 mL | 5.1 mL |
| pH * | 6.9 – 7.1 | | 7.0 | 6.9 | 6.9 | 6.9 | | 7.0 | 6.9 | 7.0 |
| Osmolality * | 270-310 mOsmol/kg | | 290 mOsmol/kg | 289 mOsmol/kg | 291 mOsmol/kg | 289 mOsmol/kg | | 289 mOsmol/kg | 290 mOsmol/kg | 291 mOsmol/kg |
| Residual solvents (DMSO) | ≤50.0 mg/L | | 15.4 mg/L | N.T. | N.T. | N.T. | | N.T. | N.T. | N.T. |
| Bacterial endotoxins | ≤5.0 EU/mL | | <0.050 EU/mL | <0.050 EU/mL | <0.050 EU/mL | <0.050 EU/mL | | <0.050 EU/mL | <0.050 EU/mL | <0.050 EU/mL |
| Sterility | Sterile | | Sterile | N.T. | N.T. | N.T. | | Sterile | N.T. | Sterile |
| UV-VIS absorption peaks * | Peaks at 280±3 nm and 676±3 nm. | | Conform | Conform | Conform | Conform | | Conform | Conform | Conform |
| Visible particles * | Essentially free of visible particles | | Conform | Conform | Conform | Conform | | Conform | Conform | Conform |
| Subvisible particles | Particles ≥10 µm ≤6000/vial  Particles ≥25 µm ≤600/vial | | ≥10 µm: 666  ≥25 µm: 9 | N.T. | N.T. | N.T. | | ≥10 µm: 381  ≥25 µm: 11 | N.T. | ≥10 µm: 3371  ≥25 µm: 50 |

N.T. denotes “not tested”. N.D. denotes “Not Detected”

**Table S5: Stability results of adalimumab-680LT technology transfer and stability batch after 18 months storage at 15-25** °**C. All tests marked with a star (*) were performed in triplicate; the mean value for each time point is shown. Of the triplicate measurements, none of the individual results fell outside the predefined specifications. All other tests were performed as single measurement or according to the applicable European Pharmacopeia method.**

|  | |  | **Test moments (months)** | | | | |  |
| --- | --- | --- | --- | --- | --- | --- | --- | --- |
| **Test** | **Specification** | | **0** | **1** | **3** | **6** | **12** | **18** |
| Protein monomer concentration * | Release: 0.95 – 1.05 mg/mL  Shelf-life: 0.90 – 1.10 mg/mL | | 1.01 mg/mL | 1.04 mg/mL | 1.02 mg/mL | 1.04 mg/mL | 1.00 mg/mL | 1.00 mg/mL |
| Protein aggregates * | Release: ≤5.0%  Shelf-life: ≤10% | | N.D. | N.D. | N.D. | N.D. | N.D. | N.D. |
| Unconjugated IRDye 680LT * | Release: ≤5.0%  Shelf-life: ≤10% | | 1.2% | 2.7% | 3.0% | 4.0% | 4.9% | 4.3% |
| Protein monomer identity * | Retention time comparable to reference standard | | Conform | Conform | Conform | Conform | Conform | Conform |
| Protein monomer integrity * | Peak shape comparable to reference standard; no shoulders, minimal tailing | | Conform | Conform | Conform | Conform | Conform | Conform |
| Target binding affinity | 50-200% | | 63% | 58% | 72% | 86% | 84% | 73% |
| Appearance (turbidity) * | Clear to slightly opalescent solution | | Conform | Conform | Conform | Conform | Conform | Conform |
| Appearance (colour) * | For information | | Ref 5 | Ref 5 | Ref 5 | Ref 5 | Ref 5 | Ref 5 |
| Container closure and label * | Closure intact, label legible and intact. | | Conform | Conform | Conform | Conform | Conform | Conform |
| Extractable volume * | ≥5.0 mL | | 5.1 mL | 5.1 mL | 5.1 mL | 5.1 mL | 5.0 mL | 5.0 mL |
| pH * | 6.9 – 7.1 | | 7.0 | 6.9 | 6.9 | 6.9 | 6.9 | 6.9 |
| Osmolality * | 270-310 mOsmol/kg | | 290 mOsmol/kg | 289 mOsmol/kg | 291 mOsmol/kg | 290 mOsmol/kg | 289mOsmol/kg | 291 mOsmol/kg |
| Residual solvents (DMSO) | ≤50.0 mg/L | | 15.4 mg/L | N.T. | N.T. | N.T. | N.T. | N.T. |
| Bacterial endotoxins | ≤5.0 EU/mL | | <0.050 EU/mL | <0.050 EU/mL | <0.050 EU/mL | <0.050 EU/mL | <0.050 EU/mL | <0.050 EU/mL |
| Sterility | Sterile | | Sterile | N.T. | N.T. | N.T. | Sterile | N.T. |
| UV-VIS absorption peaks * | Peaks at 280±3 nm and 676±3 nm. | | Conform | Conform | Conform | Conform | Conform | Conform |
| Visible particles * | Essentially free of visible particles | | Conform | Conform | Conform | Conform | Conform | Conform |
| Subvisible particles | Particles ≥10 µm ≤6000/vial  Particles ≥25 µm ≤600/vial | | ≥10 µm: 666  ≥25 µm: 9 | N.T. | N.T. | N.T. | ≥10 µm: 418  ≥25 µm: 23 | N.T. |

N.T. denotes “not tested”. N.D. denotes “Not Detected”
